# Comparison of molecular dynamics and superfamily spaces of protein domain deformation

**DOI:** 10.1186/1472-6807-9-6

**Published:** 2009-02-17

**Authors:** Javier A Velázquez-Muriel, Manuel Rueda, Isabel Cuesta, Alberto Pascual-Montano, Modesto Orozco, José-María Carazo

**Affiliations:** 1 Centro Nacional de Biotecnología-CSIC, Campus Universidad Autónoma, 28049 Madrid, Spain; 2 Molecular Modeling and Bioinformatics Unit, IRB-BSC Joint Research Program in Computational Biology, Institute for Research in Biomedicine, Josep Samitier 1-5, Barcelona 08028, Spain; 3 University of California, San Francisco, Department of Biopharmaceutical Sciences and Pharmaceutical Chemistry, 1700 4th St. UCSF/MC 2552, Byers Hall Room 503, San Francisco, CA 94158-2330, USA; 4 The Scripps Research Institute, Department of Molecular Biology, 10550 North Torrey Pines Road, Mail TPC-28, La Jolla, California, 92037, USA; 5 Departament Arquitectura de Computadores y Automática, Facultad de Ciencias Físicas, Universidad Complutense, 28040 Madrid, Spain; 6 Departament de Bioquímica i Biología Molecular, Facultat de Biología, Universitat de Barcelona, Avgda Diagonal 645, Barcelona 08028, Spain; 7 National Institute of Bioinformatics, Parc Científic de Barcelona, Josep Samitier 1-5, Barcelona 08028, Spain; 8 National Institute of Bioinformatics, Centro Nacional de Biotecnología, CSIC, Madrid, Spain; 9 Barcelona Supercomputing Center, Jordi Girona 29, Barcelona 08034, Spain

## Abstract

**Background:**

It is well known the strong relationship between protein structure and flexibility, on one hand, and biological protein function, on the other hand. Technically, protein flexibility exploration is an essential task in many applications, such as protein structure prediction and modeling. In this contribution we have compared two different approaches to explore the flexibility space of protein domains: i) molecular dynamics (MD-space), and ii) the study of the structural changes within superfamily (SF-space).

**Results:**

Our analysis indicates that the MD-space and the SF-space display a significant overlap, but are still different enough to be considered as complementary. The SF-space space is wider but less complex than the MD-space, irrespective of the number of members in the superfamily. Also, the SF-space does not sample all possibilities offered by the MD-space, but often introduces very large changes along just a few deformation modes, whose number tend to a plateau as the number of related folds in the superfamily increases.

**Conclusion:**

Theoretically, we obtained two conclusions. First, that function restricts the access to some flexibility patterns to evolution, as we observe that when a superfamily member changes to become another, the path does not completely overlap with the physical deformability. Second, that conformational changes from variation in a superfamily are larger and much simpler than those allowed by physical deformability. Methodologically, the conclusion is that both spaces studied are complementary, and have different size and complexity. We expect this fact to have application in fields as 3D-EM/X-ray hybrid models or *ab initio *protein folding.

## Background

The central dogma of structural biology asserts that the aminoacid sequence has all the information needed for a protein to adopt a structure, and that structure determines function. The connection between sequence and structure has centered a great amount of work and detailed theories of protein folding exist [1], but still predicting structure or function from sequence is a extremely complex task except in cases of high sequence identity between the target protein and a well annotated homolog [2]. There are many cases of non-homologous proteins sharing a given fold or function as well as proteins with reasonably similar sequences having quite different structures.

Flexibility seems to play an important role in protein function, as in many cases movements are key for activity. Unfortunately, still less information exists on this connection between flexibility and function and, specifically, regarding the conformational changes that need to happen in a protein to perform its biological function [3-5]. In the very same way as structures that are able to perform a specific function are conserved by evolution by not tolerating mutations that seriously modify that structure, it is plausible to think that mutations disrupting the flexibility pattern of a given protein are not going to be accepted either [3,6-9].

Inspection of structural databases such as SCOP [10], CATH [11] or FSSP [12] shows the existence of superfamilies of proteins which display very similar folds and are evolutionary related. Analysis of these superfamilies allows us to determine the structural variation within a common fold [13,14], thus defining the flexibility of that fold. Other works, using mostly coarse-grained models [14-17], suggest that, at least for some proteins, the most important deformation modes are preserved within superfamilies, supporting the idea that structural flexibility patterns tend to be conserved. However, these findings immediately raise a new question: Are flexibility patterns conserved because if disrupted, the function would be lost, or instead, are they conserved because these are the ones better tolerant to change? In more precise terms, the following two extreme scenarios, equally reasonable *a priori*, are possible:

i) If physical deformability is crucial to protein function, conformational changes introduced by sequence modifications will happen as orthogonal as possible to the physical deformation pattern.

ii) The physical deformation pattern traces movements that allow quite significant conformational changes without disruption of the function(s) associated to a fold. Mutations leading to conformational changes along this pattern of flexibility are going to be better tolerated, as they won't affect the function. This would suggest a good overlap between the physical space studied by MD and the conformational space explored by the members of a superfamily.

**Figure 1:**
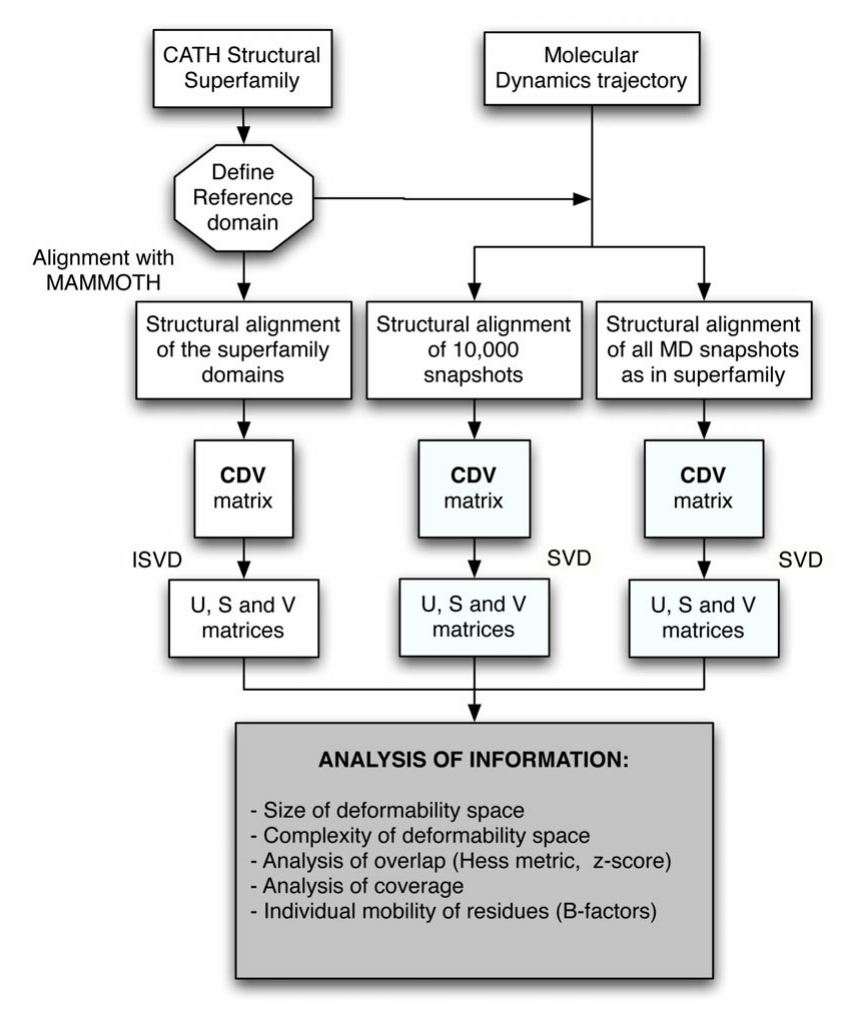
**Workflow of the comparison between SF and MD-spaces of protein domain deformability done in the study**.

Following pioneering work by Ortiz and others [18,19], here we have performed a thorough comparison of the space of protein domain flexibility shown by the members of a CATH superfamily (SF-space) with the space of protein flexibility sampled by one reference member of the superfamily by molecular dynamics (MD-space), aiming at investigating the potential overlap between both spaces and consequently, testing the possibility of using them in a combined way for applications that require protein deformation exploration. The dataset used in this work includes 55 different superfamilies selected to cover all topologies, a good distribution of domain size and presenting enough number of non-redundant members. A satisfactory reference domain to perform MD was chosen for each superfamily based on having enough sequence percentage in the core of the alignment and providing good alignments to define such as core with at least 10 members (see Methods for details). The MD-space was obtained using atomistic MD simulations in explicit water [20] and the SF-space was derived from alignment of experimental structures. Both ensembles were subjected to decomposition algorithms such as single value decomposition (SVD) and incremental singular value decomposition (ISVD), to capture and compare the essential components of their spaces (Figure 1). The use of ISVD when treating the SF-space [21] allowed us to consider regions only partially aligned within the members of the superfamily, consequently increasing the number of residues incorporated in the analysis.

Our results show that the relative flexibility among domains of a given superfamily is restricted to just a few "directions of change" (SF-space), which overlap only partially with the "directions of change" indicated by MD (MD-space). For technical purposes, the conclusion is that both spaces can be combined to increase the dimensionality of the search space when performing any kind of computational-biology task that requires the exploration of possible protein deformations.

## Results and discussion

**Figure 2:**
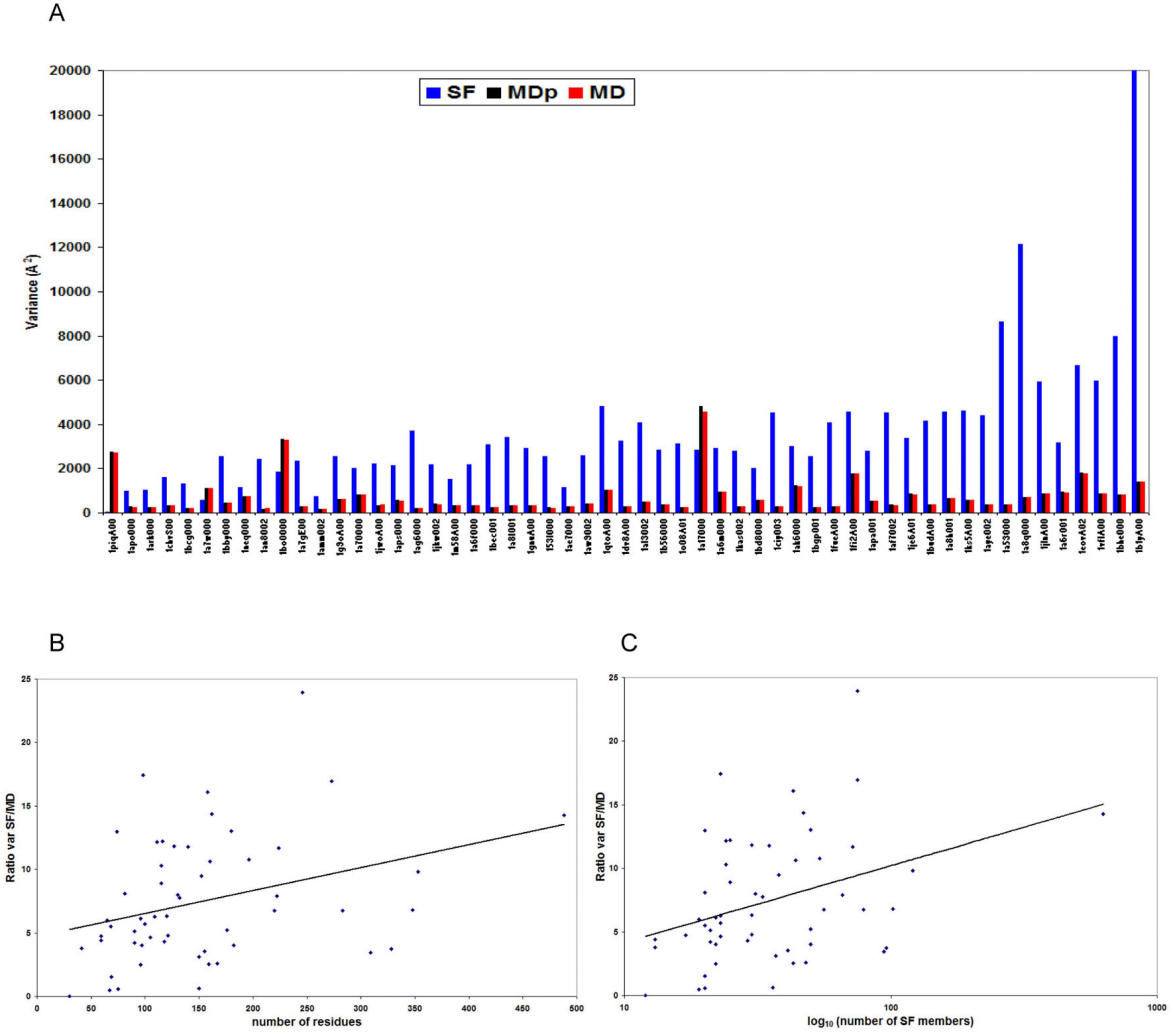
**Comparisons based on variance**. a) Total variance for the performed decompositions: ISVD of the SF-space, SVD of the partial MD-space containing as many snapshots as members in the superfamily (average values for 100 windows), and SVD of the MD-space containing the entire MD trajectory. The domains in the x-axis are sorted by increasing number of aminoacids. b) Ratio of SF- and MD-space variances against the number residues in the reference domain. c) Ratio of SF- and MD-space variances against number of superfamily  members (log scale).

To study the relative size of the MD- and SF-spaces, we computed their variance after matrix decomposition (see Figure 1) by summing the squares of all the singular values (see Methods section for details). We clearly observe that, in general, the SF-space of deformation is larger, having a variance between 2 and 25 times (in average 10 times, see Figure 2a) bigger than the MD-space. These results do not seem to be influenced by the fact that the MD-space is defined using many more structures than the SF-space, since the basic trend is kept when we restrict the calculations to a partial MD-space (named MDp) with just as many snapshots as experimental structures in the superfamily. There are only 3 cases among the 55 superfamilies analyzed in which this pattern is, without any clear reason, different (1piqA00, 1bo0000 and 1a17000). We have not found any apparent correlation between these three cases, neither structurally (they are mostly α, β and α, respectively) nor functionally (binding, enzyme, signaling). Interestingly, we do not find any relationship between the variance of the MD-space and the number of aminoacids of the domain, which can be explained considering that the factors producing more structural variability, such as flexible loops, are not affected by the size of a domain. On the contrary, the variance of the SF-space increases with the number of aminoacids of the protein (Figure 2a), which is reasonable given the linear relationship between protein length and possibilities of variation in composition through mutation. As a consequence of this different behavior of variance *versus *size, a rough increase in the ratio between SF- and MD-space variances with protein size is found (Figure 2b), and the same incremental tendency is observed for the variance ratio plotted against the number of superfamily members (Figure 2c). Again, a similar reasoning explains it: a greater size of the superfamily implies a parallel increase in the possibilities of sequence variation, while it does not affect the variance of the MD-space.

**Figure 3:**
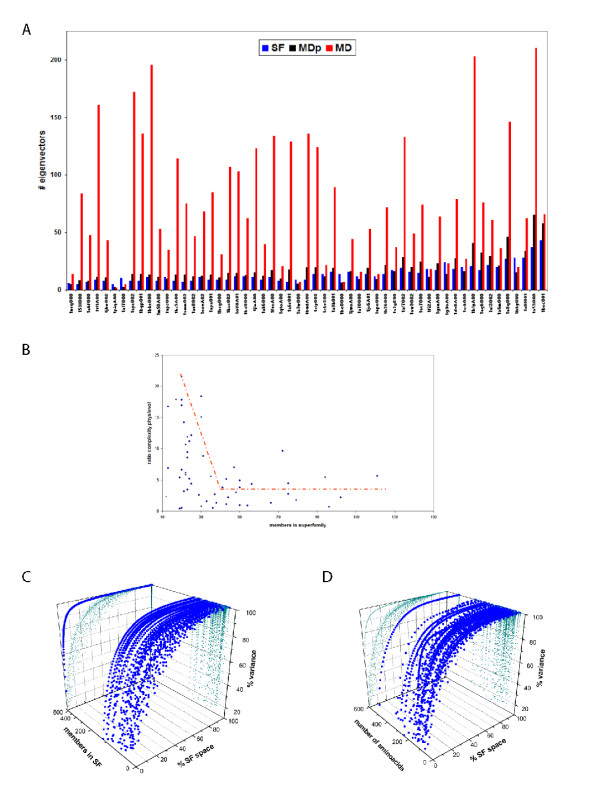
**Comparisons based on complexity**. a) Vectors required to explain 90% of the variance for the performed decompositions: ISVD of the SF-space, SVD of the partial MD-space containing as many snapshots as members in the superfamily (average values for 100 windows), and SVD of the MD-space containing the entire MD trajectory. The domains in the x-axis are sorted by increasing number of superfamily members. b) Ratio of required vectors from SF- and MD-spaces to explain 90% of the variance against the number of superfamily members. c. Cumulative variance described by the SF singular vectors versus the size of the SF-space (normalized) and the number of SF-members. d. Cumulative variance described by the SF singular vectors versus the size of the SF-space (normalized) and number of aminoacids of the domain.

Quite surprisingly, we found that the SF-space is less complex (Figure 3a) than the MD one: i.e., it requires a smaller number of singular vectors to explain a given threshold (90%) of the variance. The difference in complexity (in general a factor of 6) can be partly explained as a natural consequence of the fact that microstates that are accessible to MD are not present among the experimentally resolved structures that form a superfamily. However, when we calculate the complexity of MDp, we still see that it is larger than the complexity of SF-space (30% more), indicating that is a defined characteristic between the two spaces. As expected, the unbalance in complexity between MD- and SF-spaces generally decreases when the number of members in the superfamily increases (Figure 3b3c and 3d). However, we observe the existence of a threshold around 40–50 members after which the ratio of complexities remains approximately 3. We interpret this fact as an indication that the superfamily has saturated its possibilities to gain complexity in the MD-space with a reasonably small number of structures, in other words the "evolutionary" deformation space of the superfamily seems to be saturated rather quickly. The other types of deformation movements present in the MD trajectories seem *physically *possible, but they are not well populated within the experimental ensembles of the superfamilies, meaning that they have not been tolerated by evolution.

**Figure 4:**
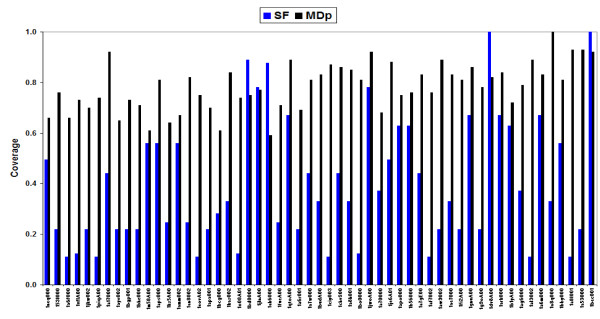
**Coverage factors for the superfamily members (SF) in the essential MD-space, and coverage factors for the partial MD-space (MDp) in the essential MD-space**. The x-axis is sorted by increasing number of members in the superfamily (the name of the reference member is written).

We employed a complementary way to analyze the ability of a superfamily to cover the MD-space, determining the coverage of its domains on the essential MD-space, the subspace defined by the first two MD singular vectors (see Methods). The results in Figure 4 show that the structures in the superfamilies do not cover well the essential MD-space, with 70% of them showing 0.5 coverage or lower, and a total average value of 0.4. The limited number of elements in the superfamilies is not responsible for this moderate coverage, since MDp covers 80% of the essential MD-space. Finally, it is worth noting that larger number of elements in the superfamily does not lead to better absolute (*versus *complete MD-ensemble) or relative (*versus *reduced MDp-ensemble) coverage (Figure 4), confirming that larger superfamilies do not necessarily sample better than the smaller ones the physical deformation space.

**Figure 5:**
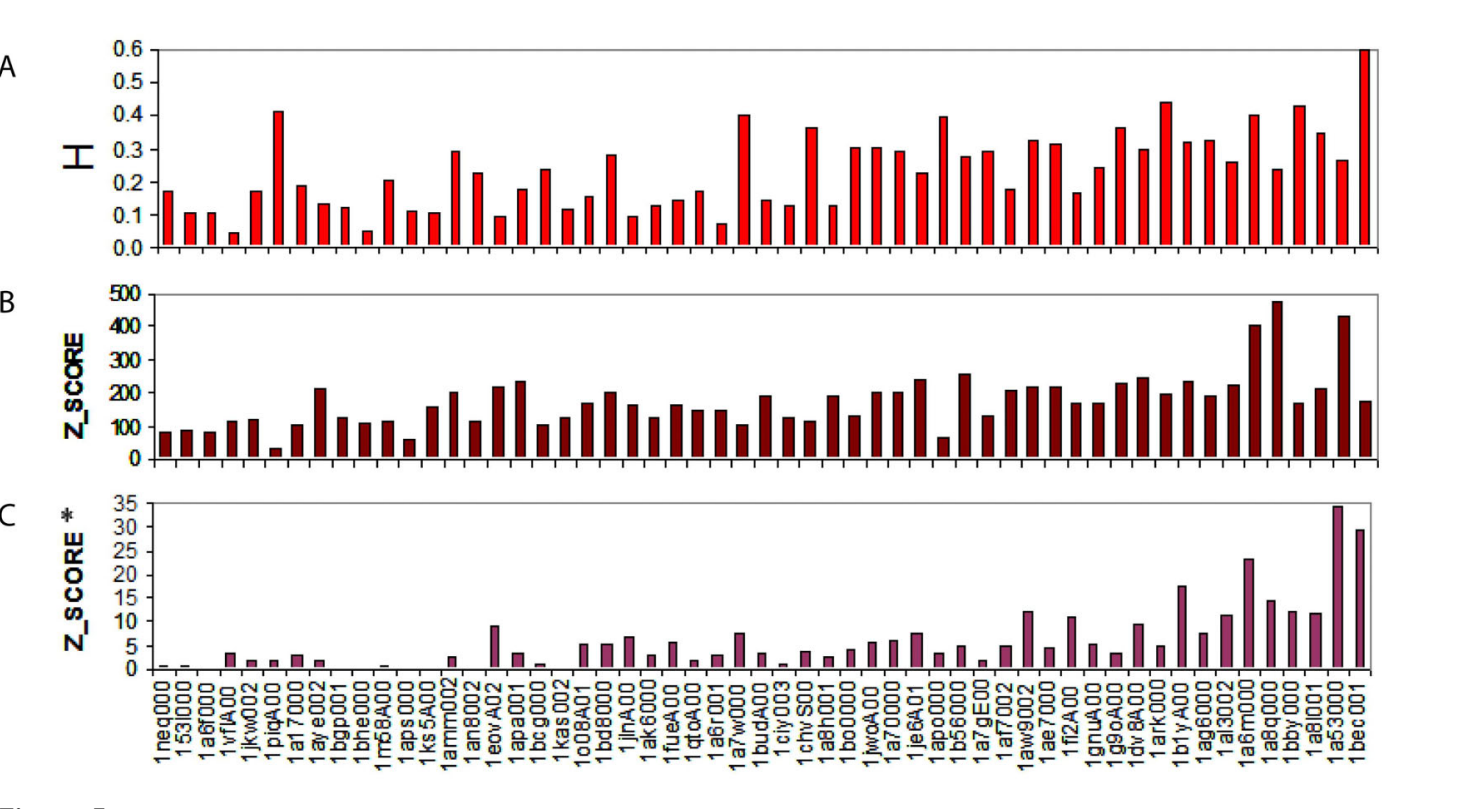
**Comparisons based on space similarity**. a) Hess metric applied using as many singular vectors as members in the superfamily. The x-axis is sorted by increasing number of members in the superfamily (the name of the reference member is written). b) Z-score of the Hess metric for a random model (See Methods for details). c) Z-score* of the Hess metric for a pseudo-random model (See Methods for details).

To study the overlap between the SF- and MD-spaces, we computed the Hess metric employing as many vectors as members in the superfamily (see Methods). In the superfamilies studied in this work, the Hess metric ranges from 0.05 to 0.6, with mean equal to 0.3 (Figure 5a). The best overlaps are found for class α and β proteins, which are explained by their simple dynamics (α) or intrinsic rigidity (β) when compared to class α+β. We found that the Hess metric values are statistically significant and not due to simple chance (see Z-scores in Figure 5b) when the results are compared to a pure random background model. Large Z-scores were also obtained when the background protein model is obtained by forcing the random trajectory to maintain covalent connectivity (Figure 5c) and to avoid steric clashes. We interpret this low, but statistically significant overlap of the SF- and MD-spaces, as a proof that proteins sharing the same fold conserve at least some part of their physical deformability pattern in order to conserve function. The rest of the deformations happening inside a superfamily by modification of the composition occur orthogonally to the deformations in the MD-space.

Putting together all the analysis commented above, we conclude that there appear to be many deformation patterns that are physically possible but are not explored within a superfamily and that the overlap between MD- and SF-spaces is only partial. The reasons for these findings could be related to the bias of the SF-space towards insertions, deletions, and changes of aminoacids leading to bigger deformations in the structure than the simple variation of the torsion angles explored in the physical space. Others reasons are probably related to the inability of the SF-space to explore movements that might challenge protein functionality.

The structural changes inside a superfamily can be severe in extension but are easily represented by a few essential movements. We cannot completely rule out the possibility that when the structures of more members of a given superfamily were solved, the overlap between spaces increased, but according to our results it seems to be an inherent limit. In summary, as suggested in the complexity analysis, the SF-space is quickly saturated.

**Figure 6:**
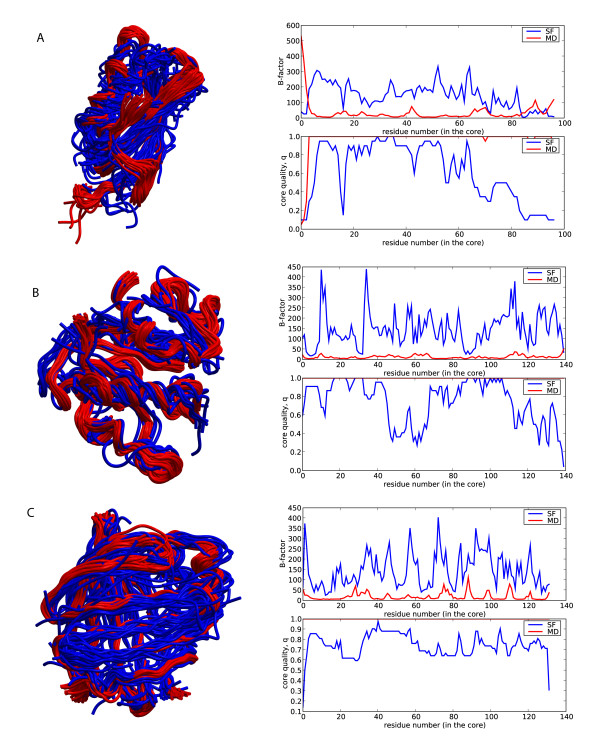
**Examples of per residue B-factor and core quality of the reference domain against the aminoacid number in the core**. The core quality *q *at a given core aminoacid is defined as the quotient of the number of times that this aminoacid has been structurally aligned and the number of superfamily members employed for the core. See Additional file 1. a) Example for superfamilies with low Hess index, H < 0.15. 1aps000. b) Example for superfamilies with n < 30 and H > 0.15. 1o08A01. c) Example for superfamilies with n > 30 and H > 0.25. 1b56000.

**Figure 7:**
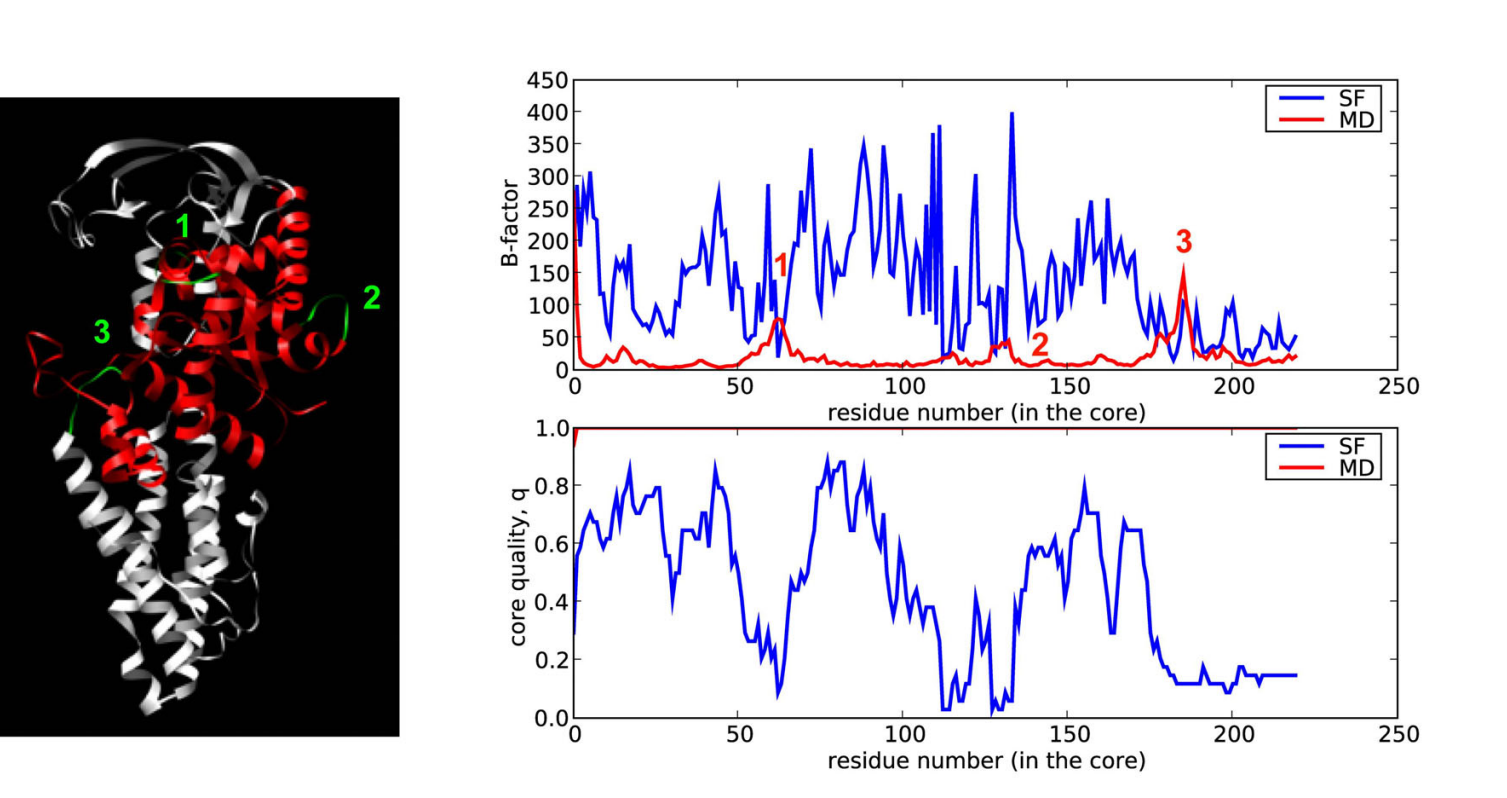
**Structure and B-factor plot for 1a8h001 (red), the anticodon-binding domain of Methionyl-tRNA synthetase from *Thermus thermophilus***. According to MD, the loops depicted in green have high flexibility, with B-factors for MD higher than those obtained from superfamily information.

**Figure 8:**
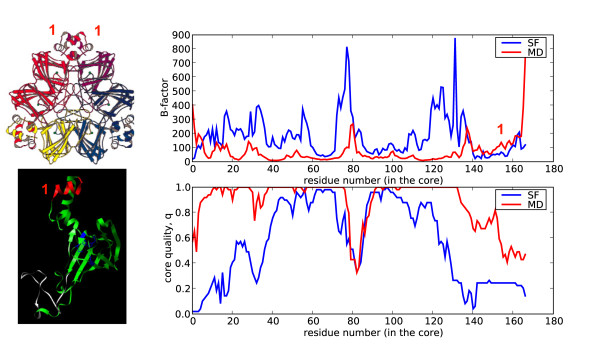
**Structure and B-factor plot for 1fi2A00 (green), oxalate oxidase from *Hordeum vulgare***. The red region (aminoacids 174–184) is involved in forming dimers as part of the final hexamer that is the active complex. In this region the B-factors are higher for MD than for the superfamily alignment.

After analyzing the global deformability patterns, we turned our attention to local residue flexibility. We computed the B-factor (see Methods) for each residue using the same data sources as before: the structural alignment of the superfamily members, and the MD trajectory of a reference domain. As expected from the previous global variance calculations, much larger B-factors are obtained from the superfamily data than from the MD trajectory (three typical cases are shown in Figure 6). Variations in sequence composition introduce dramatic local changes in a fold that would be difficult to obtain modifying the physical deformation pattern alone. We, however, observe some cases of residues with B-factors derived from MD larger than those obtained considering superfamily variation. Typically they correspond to regions involved in interactions with other macromolecules. For example, the loops (Figure 7, green) of the anticodon-binding domain of Methionyl-tRNA synthetase from *Thermus thermophilus *(1a8h001) are very flexible in our MD simulations performed in the absence of RNA, but they are frozen in the biologically-relevant RNA-bound form [22]. Similarly, the C-terminal region of Germin from *Hordeum vulgare *(1fi2A00, Figure 8, red), required for dimer formation [23], is exposed and flexible in the MD trajectory of the monomer while in the dimer the contacts trap it.

Taking into account local and global behavior together, we distinguish three groups among the 55 studied superfamilies:

i) Superfamilies (with both small and large number of members) showing poor overlap between SF- and MD-spaces (Hess index < 0.15, Additional file 1) and low correspondence between B-factor plots (Figure 6a). This group is largely enriched in enzymes of the α+β structural class. We can expect that flexibility will be a crucial issue in these proteins and accordingly the deformation pattern should be very well preserved, which means that changes in the SF-space happen as orthogonal as possible to the functionally relevant MD-space [24-26].

ii) Superfamilies with high number of members (n > 40), good overlap of SF- and MD-spaces (Hess index > 0.25, Additional file 1) and relatively good correspondence between the B-factor plots (Figure 6c). Here we find domains with structural or binding roles and fewer enzymes, with preference for α and β motives. In this group the superfamilies have been able to explore many physically-available deformation modes of the MD-space which do not interfere with function.

iii) Superfamilies with low number of members (n < 40), some overlap in the deformation spaces (Hess index > 0.15, Additional file 1) and poor correspondence between B-factor plots (Figure 6b). This group shows diverse families both in structural and functional terms. The physical deformability space has been explored to a little extent, but the residues that are not essential for function introduce large local structural changes reflected in poor B-factor correspondence.

## Conclusion

Our technical analysis comparing the spaces of structural variation within superfamilies (SF-space) and along atomistic MD simulations (MD-space) sheds light on the connection between physical flexibility and conformational variation with compositional change in the aminoacid sequence. The overall picture showed a more complex scenario than we originally thought, in part due to the fact that we are comparing a set of different structures in a SF with the MD of just one of them. First, we have observed that when the sequence of a protein changes to become another member of the superfamily, the change is produced following a way that does not completely overlap with that expected from the intrinsic physical deformability of the protein, which suggests that functional restriction limits the access to some flexibility patterns to evolution. This effect is especially clear for enzymes, where there is the worst overlap between SF- and MD-spaces. Second, our analysis shows that conformational changes resulting from sequence variation tend to be larger and much simpler than those allowed by individual physical flexibility. Interestingly, the threshold for achieving the maximum overlap between the SF and MD-spaces seems to be situated around 40 superfamily members (Figure 3b), suggesting some saturation in the deformation along the superfamily when compared to the physical space.

MD and SF spaces are comparable, but they also have important differences, and some words of caution are necessary. Since superfamily members vary in sequence, in some cases quite dramatically, and they will be expected to have different structures, while MD simulation samples the flexibility of a single sequence, it is not surprising that MD does not explain instances where there are specific chemical interactions.

The strength of our analysis relies in its interesting methodological implications. As the deformation spaces have different size and complexity and do not fully overlap, they can be considered as complementary. Flexibility analysis derived from the study of the structural variation along superfamilies can provide easy to manage and useful descriptions [21,27], although they will have a limit in the physical complexity that they can describe. In much the same way, physical descriptions of isolated domains without considering their possible interactions have a limited capability to predict their flexibility in the context of protein-protein complexes, and variation along domains in a superfamily is a good way of obtaining that information. In other words, taking together SF and MD spaces we enrich our view on the conformational freedom of proteins.

This is expected to be of especial interest in the areas of 3D-EM/X-ray hybrid models or *ab initio *protein folding, where the exploration of the physical conformational space exclusively with high dimensionality methods such as Molecular Dynamics or Normal Mode Analysis could be over-conservative. We suggest that the use of the most important singular vectors of the SF-space (about 6) will provide a complementary deformation space that can be very useful in sampling [27], since it will attract to the common fold quite distant structures. A combination of both spaces in a sequential way can help to improve these areas of protein structure prediction.

## Methods

### Superfamily space of flexibility

**Figure 9:**
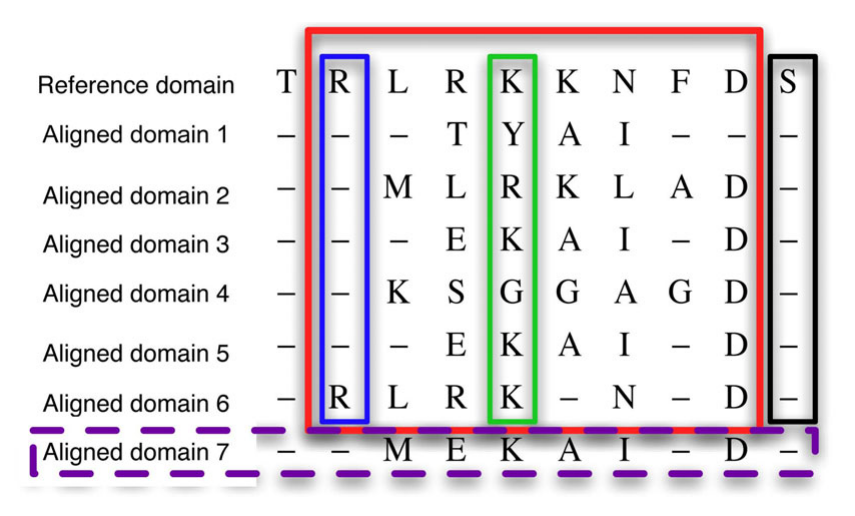
**Example of structural alignment for a superfamily**. All the domains are pairwise aligned against the reference domain. Purple discontinuous box: Domain excluded of the analysis because -ln(E) < 5. Red box: core of the alignment, composed by all the aminoacids of the reference domain aligned at least once and their correspondences. Blue box: Example of reference residue aligned with gaps (core quality: 1/6 = 17%). Green box: reference residue aligned without gaps (core quality: 6/6 = 100%). Black box: Reference residue that is not part of the core because there is not variation info for it (never aligned. Core quality 0/6 = 0%).

In order to get results from a varied and representative number of superfamilies, we looked for structural diversity, non-redundancy, and good distribution of domain size. Additionally, enough number of structures and a good percentage of the reference domain sequence length forming the core of the alignment was another selection criterion. In total, we finally selected 55 superfamilies in CATH version v3.0.0 containing at least 20 non-redundant members (redundancy defined as 95% of sequence identity or higher), belonging to all possible structural classes (α, β, α+β), and with a good span in sequence size (30–459 aa). The decomposition of the conformational space defined by a given superfamily was done following the same approach developed for flexible fitting in tridimensional electron microscopy (3D-EM) in the presence of incomplete data [21]. All the domains of the superfamily were structurally aligned using MAMMOTH [28] against the reference domain, that was studied with MD (Figure 1). The domains with a statistical significance score of -ln(E) > 5 as provided by MAMMOTH where used to build the core of the structural alignment for the superfamily (red box, Figure 9), being the rest excluded (purple discontinuous domain, Figure 9). The condition for an aminoacid of the reference domain to be part of the core is to be aligned at least once with the rest of the superfamily members (example in blue box, Figure 9). The 55 superfamilies selected for this study had at least 10 domains and 68% of the reference domain sequence length belonging to the core, with most of them showing even a higher value (90%), thus providing data with as least missing values as possible.

Once the domains were aligned, the coordinates of the aminoacids in the core were used to build the coordinate displacement vectors (*cdv's*):

(1) $$cdv_{i}=(x_{j}^{n}-x_{i}^{n},y_{j}^{n}-y_{i}^{n},z_{j}^{n}-z_{i}^{n})$$

where *x*, *y*, *z *stand for the coordinates of the same backbone atom *n *(C_α_, O, N and C) in two structurally aligned aminoacids, each one belonging to one domain (*i *for the reference, *j *for the aligned). A CDV vector was created by using all the *cdv's *obtained for the atoms of a given aligned domain, placing *x*, *y*, *z *coordinates in consecutive indexes. Then a **CDV **matrix was built with all the CDVs as its columns (one per aligned domain). The **CDV **matrix was decomposed with the incremental singular value decomposition (ISVD) algorithm [29] to capture the main axes of variation (Figure 1). The use of ISVD, a variant of the single value decomposition (SVD) method [30], allows us to manage superfamilies with incomplete information in the core due to gaps in the alignment, since it can handle matrices for which some of the values of their elements are unknown. In any case, aminoacids in the reference domain that cannot be aligned in any of the pairwise alignments using MAMMOTH (black box, Figure 9) were excluded of further analysis. When ISVD is applied to the **CDV **matrix it produces:

(2) **CDV **= **U**·**S**·**V^T^**

**U **- 4*3*m × *n-1 *matrix containing an orthogonal basis for the multi-dimensional space defined by the CDVs, were *m *is the number of aminoacids in the core and *n *is the number of superfamily members used in the procedure. 4 comes for the 4 backbone atoms employed and 3 comes from the x, y, z coordinates.

**S **- *n-1 *× *n-1 *diagonal matrix containing the *n-1 *singular values of the decomposition.

**V **- *n-1 *× *n-1 *matrix containing an orthogonal basis for the space of the rows of **CDV**.

The elements of the columns of **U **define a new basis for **CDV **which, ranked by the relative value of the singular values in **S**, best explains the structural variation among the aligned domains. The ISVD algorithm estimates the incomplete columns of the original **CDV **matrix during the decomposition procedure in an incremental fashion, starting with the columns with less missing values. If the next CDV vector **c **has missing values, denoted as **c**_0_, they are estimated by:

(3) $$c_{0}={U^{\prime }}_{0}\cdot S^{\prime }\cdot Z$$

where **Z **is the set of values that minimize the sum of squared errors for the known values, denoted as **c**_•_, when solving:

(4) $${U^{\prime }}_{•}\cdot S^{\prime }\cdot Z=c_{•}$$

In eqs. (3) and (4), ${U^{\prime }}_{0}$ and ${U^{\prime }}_{•}$ are the corresponding rows of **U' **for the missing and known data, respectively. **U' **and **S' **are the decomposition matrices calculated in intermediate steps of the ISVD procedure. The interested reader is referred to [29] for the theory behind the ISVD, and to [21] for a complete explanation of the adaptation of ISVD to structural alignments of superfamilies. As in Principal Component Analysis (PCA), the result of both SVD and ISVD calculations is a transformation of the initial variation matrix into a set of orthogonal movements characterized by a set of singular vectors (which indicates the nature of the essential movement) and a set of singular values which, after transformation by eq. 5, are equal to the PCA eigenvalues.

(5) $$l_{i}=n\cdot s_{i}^{2}$$

where *n *is the number of snapshots used for the decomposition, *l*_*i *_is the PCA eigenvalue and *s*_*i *_is the [I]SVD singular value. Note that the original protein Cartesian coordinates appear now as projections onto the space defined by the singular vectors without any loss of structural information.

### Molecular-dynamics space of flexibility

The range of conformations accessible for a protein under normal physiological conditions can be well explored by molecular dynamics (MD) simulations. The technique samples the movements of macromolecules by integration of Newton equations of motion, with the forces being obtained from an accurate potential functional (the force field) fitted to reproduce high accurate quantum mechanical data in small model systems [31,32]. In opposition to Normal Mode Analysis, atomistic MD does not assume that the protein should be confined in a harmonic well around the experimental structure, allowing then, if required by the physics of the system, large conformational transitions. It is the best technique to explore the physical deformation space for proteins.

The reference protein domains were simulated in the context of the whole native protein. All protein structures were titrated, neutralized by ions, minimized, hydrated, heated and equilibrated (for at least 0.5 ns) using a well established protocol [20]. Trajectories were collected using AMBER parm99 force field [33] in conjunction with Jorgensen's TIP3P model [34,35] for representing water molecules. Particle Mesh Ewald approach was used to deal with long-range effects [36]. Integration of motion equations was performed every 1 fs, the vibrations of bonds involving hydrogen atoms being removed by SHAKE algorithm [37]. Production runs were obtained with the program AMBER8 [38] and were extended for 10 ns. Computational effort performed here corresponds to more than 20 CPU years and were done thanks to access to large supercomputer resources.

### Statistical descriptors for comparison

The MD and SF-spaces were subjected, for comparison purposes, to a modified version of the essential dynamics procedure [39] using SVD (with MD-space) and ISVD (with SF-space) decompositions. Many comparisons can be easily made using the singular vectors and values provided by the decomposition algorithms:

*1) The size of deformability space *was measured by the variance in MD or superfamily ensembles, summing the square of the singular values obtained after the decomposition. To avoid bias related to the limited number of structures in most superfamilies, the analysis of MD variance was repeated also using as many equally spaced MD snapshots as superfamily members (partial-MD space; MDp). The average values for 100 windows were computed.

*2) The complexity of the deformability space *was determined by the number of singular vectors needed to explain 90% of the variance.

*3) The overlap between the SF- and MD-spaces *was determined using the Hess metric [40] and associated Z-score (eqs. 6 and 7; [41]).

(6) $$H=\frac{1}{n}\sum_{i=1}^{n}\sum_{j=1}^{n}{\left(u_{i}^{X}•u_{j}^{Y}\right)}^{2}$$

where *X *and *Y *stand for the two methods, the indexes *i *and *j *stand for the orders of the eigenvectors (ranked according to their contribution to the structural variance), and *n *stands for the number of superfamily members.

(7) $$Z_{score}=\frac{\left(H(observed)\right)-\left(H(random)\right)}{std\left(H(random)\right)}$$

Pure random models were obtained by decomposition of a pseudo-covariance matrix obtained by random permutation of the backbone atoms for each snapshot in a trajectory, and the standard deviation (std) was obtained by considering 500 different pseudo-covariance matrices.

Additional Z-scores* (labeled with * to avoid confusion with previous Z-scores derived from purely random models) showing the relevance of the values for H in a more chemically sound environment were computed from models where the chemical connectivity was maintained and steric collapses were avoided. For this purpose, we performed several 10 ns discrete dynamics simulations for each protein with a simplified force-field defined by covalent bonds plus a hard sphere potential for each atom [42]. Essential dynamics from these trajectories provided sets of singular vectors being representative from random movements but still consistent with the basic physics of the protein. The standard deviations needed for Z-score calculations were evaluated from independent discrete dynamics simulations.

**Figure 10:**
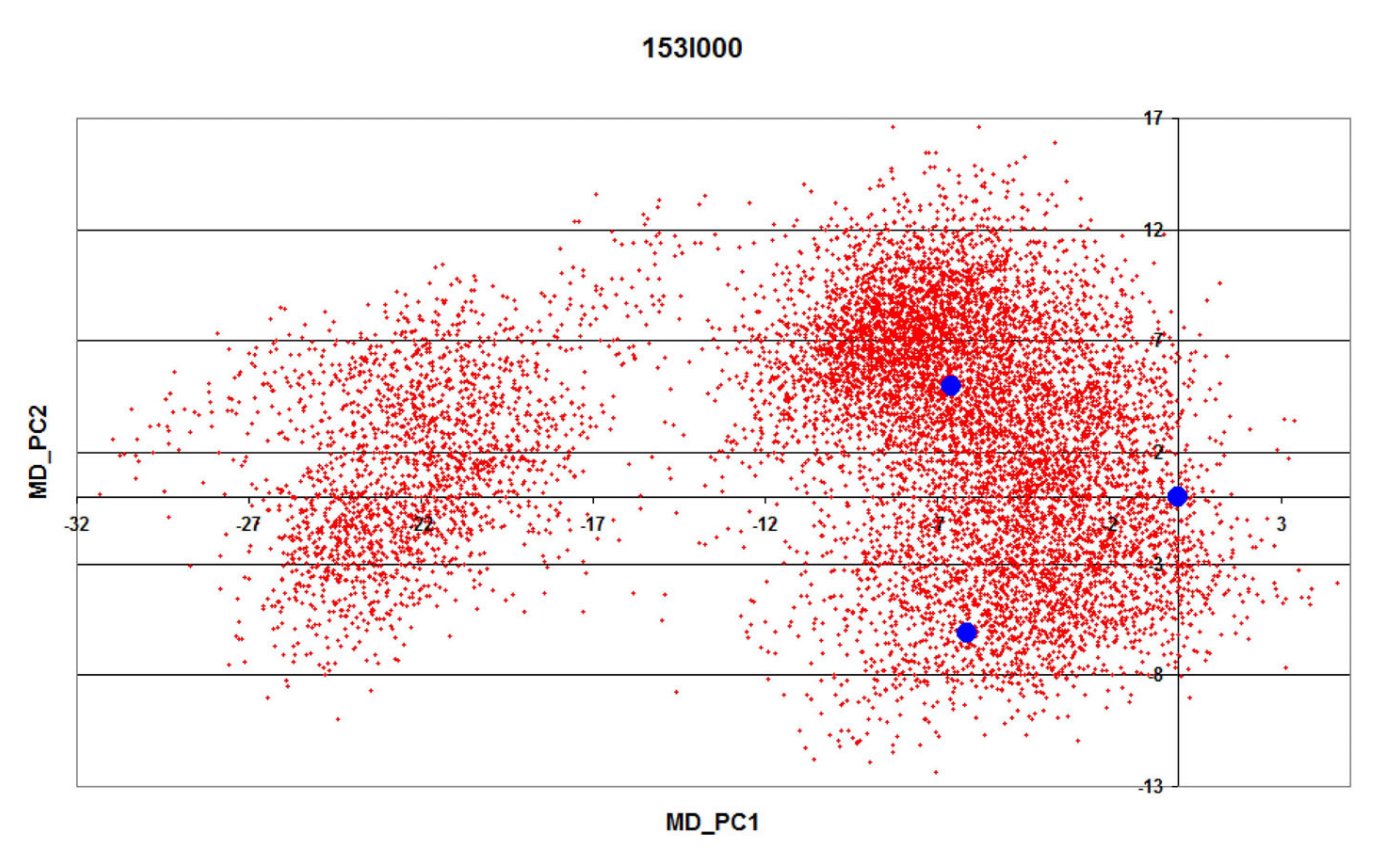
**Example of coverage of the essential MD-space achieved by SF-space**. The limits of the essential MD-space were determined by the smallest and largest projection values achieved during 10 ns trajectories (10000 structures, red). The essential MD-space was divided in 9 equivalent portions and coverage was evaluated as the number of portions of the essential MD-space visited by at least one superfamily structure (blue).

*4) The coverage of MD-space achieved by the SF-space *was measured by analyzing the distribution of the projections of the superfamily members on the essential subspace defined by the two first singular vectors of the MD-space (essential MD-space). The essential MD-space was divided into 9 equivalent portions were the maximum X and Y values were determined by the smallest and largest projection values achieved during the 10 ns trajectories. The coverage was evaluated as the number of portions of the MD-essential space that were visited by at least one superfamily member (example in Figure 10). Similar results were obtained changing the number of portions. Note that a low coverage can obey to the intrinsic differences between MD and superfamily-derived samplings, but also to the limited number of superfamily members available. In order to distinguish between both sources of deviation we also computed the coverage for the partial MD-space.

*5) Individual mobility of residues *was determined by the residue B-factors:

(8) $$\text{B=}\frac{\text{8}}{\text{3}}\pi^{\text{2}}\langle \Delta r^{2}\rangle$$

where ⟨Δ*r*^2^⟩ stands for the oscillations of atoms around equilibrium positions.

Due to the fact that the structural alignment of the superfamilies yields incomplete sets of coordinates, we applied a Metropolis Monte Carlo algorithm with a Hamiltonian method [41] which allowed us to obtain energetically permitted projections along each singular vector within the SF-space (see eq. 9). The displacements obtained can then be projected to generate Cartesian "pseudo-trajectories" which have complete coordinates and are representative of the superfamily ensemble. The B-factors can be easily obtained from this pseudo-trajectory.

(9) $$E_{X}=\sum_{i=1}^{n}k_{i}^{X}\Delta D_{i}^{X}$$

where *n *is the number of superfamily members and $\Delta D_{i}^{X}$ stands for a displacement along a given mode (*i*) in the space *X*. $k_{i}^{X}$ is the stiffness constant associated with a deformation mode, computed as *k*_*b*_*T*/(2*l*_*i*_), with k_b _being Boltzmann's constant, *l*_*i *_the corresponding PCA eigenvalue and T the absolute temperature.

## Competing interests

The authors declare that they have no competing interests.

## Authors' contributions

JAVM designed the experiments, performed the analysis of superfamily and MD data with SVD/ISVD algorithms, analyzed results and wrote the manuscript. MR designed the experiments, provided the MD simulations, compared the SF and MD-spaces, analyzed results and wrote the manuscript. JAVM and MR should be considered at the same level of authorship. IC analyzed results, obtained the biological examples, and revised the manuscript. APM analyzed results and provided help and discussion with decomposition algorithms. MO and JMC designed experiments, analyzed results and wrote the manuscript. All authors read and approved the final manuscript.

## Supplementary Material

Additional file 1 **Table of the domains and superfamilies employed for this study.** Table of the domains and superfamilies employed for this study, with their function and structural class. The domains are sorted by the similarity between the SF- and MD-spaces according to the Hess metric. Click here for file
