# Supplementary material for: Comparison of molecular dynamics and superfamily spaces of protein domain deformation
Source: BMC Struct Biol. 2009 Feb 17;9:6. doi: 10.1186/1472-6807-9-6 (PMC2666742; doi:10.1186/1472-6807-9-6)
Supplement: Additional file 1 — Table of the domains and superfamilies employed for this study. Table of the domains and superfamilies employed for this study, with their function and structural class. The domains are sorted by the similarity between the SF- and MD-spaces according to the Hess metric. [file 1472-6807-9-6-S1.doc]

Table S 1

| **Domain** | **C** | **A** | **T** | **H** | **Elements in CATH superfamily** | **MAMMOTH condition -ln(E)** | **Members in the core** | **Class** | **Function** | **Function Details** | **Hess Metric** |
| --- | --- | --- | --- | --- | --- | --- | --- | --- | --- | --- | --- |
| 1vflA00 | 3 | 20 | 20 | 140 | 27 | -5 | 17 | α+β | Enzyme | Transferase | 0.04 |
| 1bhe000 | 2 | 160 | 20 | 10 | 20 | -5 | 20 | β | Enzyme | Hydrolase | 0.05 |
| 1a6r001 | 3 | 90 | 70 | 10 | 34 | -5 | 30 | α+β | Enzyme | Peptidase | 0.07 |
| 1jlnA00 | 3 | 90 | 190 | 10 | 24 | -5 | 24 | α+β | Enzyme | Tyrosine phosphatase | 0.09 |
| 1eovA02 | 3 | 30 | 930 | 10 | 25 | -5 | 22 | α+β | Enzyme | Ligase (Nucleic acid binding) | 0.09 |
| 1ks5A00 | 2 | 60 | 120 | 180 | 21 | -5 | 21 | β | Enzyme | Hydrolase | 0.10 |
| 1a6f000 | 3 | 30 | 230 | 10 | 31 | -5 | 13 | α+β | Enzyme | Endonuclease | 0.10 |
| 153l000 | 1 | 10 | 530 | 10 | 27 | -5 | 13 | α | Enzyme | Hydrolase (O-glycosyl) | 0.11 |
| 1aps000 | 3 | 30 | 70 | 100 | 21 | -5 | 21 | α+β | Enzyme | Acetilphosphatase | 0.11 |
| 1kas002 | 3 | 40 | 47 | 10 | 25 | -5 | 23 | α+β | Enzyme | Acyltransferase, fatty acid elongation | 0.12 |
| 1bgp001 | 1 | 10 | 520 | 10 | 20 | -5 | 20 | α | Enzyme | Peroxidase | 0.12 |
| 1ak6000 | 3 | 40 | 20 | 10 | 26 | -5 | 25 | α+β | Binding | Actin depolymerizing Factor | 0.13 |
| 1a8h001 | 3 | 40 | 50 | 620 | 68 | -5 | 35 | α | Enzyme | tRNA ligase | 0.13 |
| 1ciy003 | 2 | 60 | 120 | 260 | 43 | -5 | 31 | β | Binding | Binding cellular surface receptor | 0.13 |
| 1aye002 | 3 | 40 | 630 | 10 | 27 | -5 | 20 | α+β | Enzyme | Exopeptidase | 0.13 |
| 1fueA00 | 3 | 40 | 50 | 360 | 26 | -5 | 25 | α+β | Electron transfer | Flavodoxin | 0.15 |
| 1budA00 | 3 | 40 | 390 | 10 | 38 | -5 | 30 | α+β | Enzyme | Metaloprotease | 0.15 |
| 1o08A01 | 3 | 40 | 50 | 1000 | 23 | -5 | 23 | α+β | Enzyme | hydrolase | 0.16 |
| 1fi2A00 | 2 | 60 | 120 | 10 | 56 | -5 | 50 | β | Enzyme | As hexamer | 0.16 |
| 1qtoA00 | 3 | 10 | 180 | 10 | 31 | -5 | 29 | α+β | Enzyme | Deoxigenase | 0.17 |
| 1jkw002 | 1 | 10 | 472 | 10 | 20 | -5 | 19 | α | Cyclin | Cyclin, cell cycle, cell division | 0.17 |
| 1neq000 | 1 | 10 | 260 | 40 | 21 | -5 | 12 | α | Binding | DNA binding protein | 0.17 |
| 1apa001 | 3 | 40 | 420 | 10 | 23 | -5 | 23 | α+β | Binding | antiviral protein. Ribosome inactivating | 0.17 |
| 1af7002 | 3 | 40 | 50 | 150 | 66 | -5 | 47 | α+β | Enzyme | Methyltransferase, chemotaxis receptor methylation | 0.18 |
| 1a17000 | 1 | 25 | 40 | 10 | 21 | -5 | 20 | α | Enzyme | Serine/threonine protein phosphatase 5 | 0.19 |
| 1m58A00 | 3 | 10 | 130 | 10 | 20 | -5 | 20 | α+β | Enzyme | Hydrolase. RC-RNase2 ribonuclease | 0.20 |
| 1an8002 | 2 | 40 | 50 | 110 | 22 | -5 | 22 | β | Toxin | Bacterial SuperAntigen | 0.23 |
| 1je6A01 | 3 | 30 | 500 | 10 | 42 | -5 | 41 | β | Antigen recognition | MHC class I chain-related protein. (MHC antigen-recognition domain) | 0.23 |
| 1a8q000 | 3 | 40 | 50 | 1820 | 100 | -5 | 94 | α+β | Enzyme | Bromoperoxidase | 0.24 |
| 1bcg000 | 3 | 30 | 30 | 10 | 35 | -5 | 23 | α+β | Toxin | Binding to Na-channels | 0.24 |
| 1gnuA00 | 3 | 10 | 20 | 90 | 52 | -5 | 50 | α+β | Transport | Gamma-aminobutyric acid receptor-associated protein | 0.24 |
| 1al3002 | 3 | 40 | 190 | 10 | 122 | -5 | 75 | α+β | Cofactor | Cys regulon transcriptional activator | 0.26 |
| 1a53000 | 3 | 20 | 20 | 70 | 129 | -5 | 121 | α+β | Enzyme | Aldolase | 0.26 |
| 1b56000 | 2 | 40 | 128 | 20 | 58 | -5 | 43 | β | Binding | Binding Fatty-Acids | 0.27 |
| 1bd8000 | 1 | 25 | 40 | 20 | 24 | -5 | 24 | α | Inhibitor | Tumor suppressor, cdk4/6 inhibitor | 0.28 |
| 1amm002 | 2 | 60 | 20 | 10 | 22 | -5 | 22 | β | Structural | Eye lens protein, crystallin | 0.29 |
| 1a7gE00 | 3 | 30 | 70 | 330 | 55 | -5 | 44 | α+β | Binding | DNA binding domain | 0.29 |
| 1a70000 | 3 | 10 | 20 | 30 | 45 | -5 | 38 | α+β | Enzyme | Ferredoxin | 0.29 |
| 1dv8A00 | 3 | 10 | 100 | 10 | 59 | -5 | 56 | α+β | binding | Carbohydrate recognition domain | 0.29 |
| 1bo0000 | 2 | 40 | 50 | 40 | 41 | -5 | 36 | β | Signaling | Chemotactic cytokine | 0.30 |
| 1jwoA00 | 3 | 30 | 505 | 10 | 37 | -5 | 37 | α+β | Enzyme | Transferase | 0.31 |
| 1ae7000 | 1 | 20 | 90 | 10 | 52 | -5 | 50 | α | Enzyme | Hydrolase, phospholipase a2, lipid degradation. | 0.32 |
| 1b1yA00 | 3 | 20 | 20 | 80 | 113 | -5 | 72 | α+β | Enzyme | Amilase | 0.32 |
| 1ag6000 | 2 | 60 | 40 | 420 | 88 | -5 | 75 | β | Electron transport | Copper binding. Plastocyanin | 0.33 |
| 1aw9002 | 1 | 20 | 1050 | 10 | 48 | -5 | 48 | α | Enzyme | Tranferase | 0.33 |
| 1a8l001 | 3 | 40 | 30 | 10 | 128 | -5 | 102 | α+β | Enzyme | Oxidoreductase | 0.34 |
| 1chvS00 | 2 | 10 | 60 | 10 | 36 | -5 | 33 | β | Toxin | Toxin(snake) | 0.36 |
| 1g9oA00 | 2 | 30 | 42 | 10 | 58 | -5 | 54 | β | Signaling | NHE-RF. Trans-membrane | 0.36 |
| 1apo000 | 2 | 10 | 25 | 10 | 63 | -5 | 43 | β | Blood coagulation | Laminin. Coagulation factor | 0.40 |
| 1a6m000 | 1 | 10 | 490 | 10 | 80 | -5 | 79 | α | Structural | Globin | 0.40 |
| 1a7w000 | 1 | 10 | 20 | 10 | 31 | -5 | 30 | α | Structural | Histone | 0.40 |
| 1piqA00 | 1 | 20 | 5 | 170 | 22 | -4 | 19 | α | Binding | DNA binding protein | 0.41 |
| 1bby000 | 1 | 10 | 10 | 10 | 142 | -5 | 96 | β | Tx factor | Winged helix | 0.43 |
| 1ark000 | 2 | 30 | 30 | 40 | 67 | -5 | 66 | β | binding | Transferase. SH3 domain of NEBULIN | 0.44 |
| 1bec001 | 2 | 60 | 40 | 10 | 915 | -10 | 628 | β | Receptor | Beta chain of a t cell antigen receptor | 0.60 |
